# Review of the Use of Entropy to Understand the Thermodynamics of Pure-Substance PCMs

**DOI:** 10.3390/e27111130

**Published:** 2025-10-31

**Authors:** Harald Mehling

**Affiliations:** Consultant (R&D), Weingartenstr. 37, 97072 Würzburg, Germany; harald.mehling@gmail.com

**Keywords:** phase change material, PCM, enthalpy, temperature, entropy, element, compound, particle, interaction, bond

## Abstract

Materials that allow the storage of a significant amount of heat in a narrow temperature range by a solid–liquid or a solid–solid phase change are called Phase Change Materials (PCMs). Understanding the thermodynamics of PCMs is crucial in PCM R&D for identifying candidate materials, developing new PCMs, and optimizing known PCMs. In this work, a review of the use of entropy to understand the thermodynamics of pure substances as PCMs is performed. Among pure substances, water, alkanes, alkanols, and fatty acids are well-known. Because they give valuable information, elements are also included. While phase change enthalpy and temperature are easy to comprehend and are directly used for application, the opposite holds for entropy. Thus, entropy usually receives little attention. However, as this review shows, entropy is of central importance, and even if it is not analyzed explicitly, then it is implicitly included in the data. If explicitly used, it can reveal crucial information. This is shown by a review of analysis tools and their results from analyzing typical PCMs. The review shows that if entropy is used systematically, a significant improvement in the understanding of the thermodynamics of PCMs is possible.

## 1. Introduction

### 1.1. Background on Phase Change Materials (PCMs)

Thermal energy storage (TES), commonly also called heat or cold storage depending on the application, has a crucial role in the energy system. It shifts, or just buffers, the supply as well as the demand for heat or cold. Historically, four different options for TES have been distinguished and are ideally based on well-defined effects. The simplest one is that a material does not undergo any change; it just changes its temperature more or less in proportion to the heat supplied or withdrawn. The heat is then called sensible heat, as the associated temperature change can be sensed, and the option for TES is accordingly called sensible heat storage. Next are phase changes between solid and liquid or different solid phases, which typically occur in a narrow temperature range. If the phase change is at a single temperature, the heat is also called latent heat, as it cannot be sensed without a temperature change. The option for TES is called latent heat storage. Materials that store a significant amount of heat in a narrow temperature range by a solid–liquid or solid–solid phase change are called Phase Change Materials (PCMs), often also latent heat storage materials (even if some temperature change is involved). Phase changes involving a gas phase, e.g., liquid–gas, require a large container to store the gas phase, or they require the use of a gas that can be stored in the atmosphere and later retrieved; thus, in the latter case, the gas must be a natural part of the atmosphere (e.g., O_2_ or H_2_O). As these gases do not condense at ambient conditions, another solid or liquid substance is needed to absorb them and to close the heat storage cycle. Commonly, the term sorption heat storage is used, subdivided into physi- and chemi-sorption depending on the effect of binding the gas. The last option is heat storage with a reversible chemical reaction. It is not clearly separated from chemi-sorption, and both are also called thermo-chemical heat storage. The four TES options are generally not a consistent classification as they refer to different issues: the heat effect in the material (chemical, etc.), a process (sorption), or if heat is stored with or without a temperature change (sensible or latent). Following the definition of PCMs given above, chemical processes are also not excluded in PCMs. None are present in most PCMs (e.g., alkanes), but concentration changes (a chemical process) are not uncommon in mixtures (e.g., salt hydrates), and most importantly, recent research has even identified PCMs with chemical reactions.

Due to the ability of PCMs to store a significant amount of heat in a narrow temperature range, PCMs are preferably used for TES with high storage density (per mass or volume) in a small temperature range and for passive temperature stabilization (temperature control). Today, a wide range of PCMs are used commercially in many applications, and for many more potential applications, intense R&D is ongoing. The most common PCM is water, with a phase change temperature *T*_pc_ between solid and liquid at 0 °C and an enthalpy change in phase change Δ_pc_*H* of 333 kJ/kg or 306 kJ/L (at the lowest density of 0.92 kg/L). For other application temperatures, other materials with suitable *T*_pc_ have to be used. They are found among pure substances, e.g., water, alkanes, fatty acids, alcohols, and salts, and non-pure substances (thus mixtures), e.g., eutectic mixtures of water with salts, salt hydrates, or mixtures of alkanes, salts, etc.

To identify candidate materials, develop new PCMs, and optimize known PCMs, an understanding of the thermodynamics of PCMs is needed. Most important are the enthalpy change in phase change Δ_pc_*H* and the phase change temperature *T*_pc_. The kinetics of phase change and other issues are also interesting.

### 1.2. Basic Thermodynamics of Phase Changes—Macroscopic and Microscopic

In thermodynamics, looking at phases and phase changes, the macroscopic description of bulk materials is commonly by their temperature *T*, pressure *p*, enthalpy *H*, entropy *S*, and often, Gibbs energy *G*. In general, a change from one phase to another is related to a change in enthalpy *H* and also entropy *S*. The enthalpy *H* describes the state regarding energy and its change, while the entropy *S* describes the state regarding disorder and its change (e.g., it determines whether a phase change is reversible or irreversible). If two phases of a material coexist, in equilibrium at temperature *T* and pressure *p*, then

Δ*G* = 0. (1)

Using the definition of Gibbs energy *G* gives

∆*G* = ∆*H* − ∆(*T*∙*S*) = 0, (2)

and since both phases are in equilibrium at the same temperature *T*, it follows that

∆*H* = *T*∙Δ*S*. (3)

On a microscopic level, enthalpy *H* and entropy *S* are determined by the relative position and motion of the particles, thus atoms or molecules, and for molecules also the position and motion of the atoms they are made of. However, it is in different ways. A change in the enthalpy *H*, being an energy, is in a simple manner related to the interactions (usually electromagnetic) as potential energy and the motions as kinetic energy. A change in the entropy *S*, which describes disorder, is more complicated related to the number of possible states *Ω*, as (k = 1.38∙10^−23^ J/K is the Boltzmann constant)

*S* = k∙ln(*Ω*). (4)

Compared to the enthalpy *H* and its change, the entropy *S* is more abstract, and its change is more difficult to comprehend. As a consequence, and because of the direct importance of Δ_pc_*H* and *T*_pc_ for applications, the entropy *S* is commonly receiving less attention in PCM R&D. However, Equation (3) already shows that the entropy connects the two most important values, Δ_pc_*H* and *T*_pc_; thus, it must contain important information. Specifically, looking at materials for application in a given *T*_pc_ range, the higher Δ_pc_*S*, the larger Δ_pc_*H*.

### 1.3. Goal, Scope, and Structure of the Review

Understanding the thermodynamics of PCMs is crucial in PCM R&D to identify candidate materials, develop new PCMs, and optimize known PCMs. In this review, the potential of entropy to contribute and its current state and use are analyzed. It covers the work conducted, the crucial results, and the knowledge gained. The focus is on the enthalpy change in phase change Δ_pc_*H* and the phase change temperatures *T*_pc_, with less on other issues like the kinetics of phase change, etc. The scope is pure substances (elements and chemical compounds). Currently, there seems to be no literature on the use of entropy to understand the thermodynamics of non-pure substance PCMs. The review is structured by the detail of analysis, starting with a graphical analysis of bulk material data to identify trends and correlations and, thus, materials that probably have underlying effects in common. Further steps involve the particles and their interactions, the phases involved, and finally simulations on an atomic and molecular level.

## 2. Materials and Methods

### 2.1. Origin and Accuracy of Values

The phase change enthalpy Δ_pc_*H* and the phase change temperature *T*_pc_ can be experimentally determined by calorimetric measurements. The phase change entropy Δ_pc_*S* is, however, not experimentally accessible; it can be calculated via Equation (3) using available data of the phase change enthalpy and temperature.

Regarding the accuracy of values, relevant for analysis, it is necessary to keep several issues in mind. At best, the uncertainty in Δ_pc_*H* is some 2%, that in *T*_pc_ is some 0.1 K and, thus, e.g., at 300 K about 0.03%. However, such accuracy is not achieved in most measurements, and this has a wide range of reasons. Most obvious is the accuracy of the calorimeter used, with regard to heat and temperature measurements. Often instrument specifications are reported, without verification by testing standard calibration materials. A major problem is also the temperature resolution, *H*(*T*). It is generally known that continuous heating or cooling of a sample causes an internal temperature gradient, thus a distortion and shift in *H*(*T*) curves. It is well-known that this effect is specifically strong in PCMs. Still, often, far too high heating and cooling rates are used, and their effect on the result is not even tested. A negative side effect of this is that close phase transitions might not be resolved as independent and instead look like a single phase transition. Mixtures might not even have a single phase change temperature, e.g., those with a peritectic transition where the phase transition takes place in a temperature range. Further, if impurities are present, then melting typically starts somewhat earlier than is the case for the material without any impurities. Thus, different ways are used to read the melting temperature from a measured *H*(*T*) curve, whether referring to the substance tested or to what it would have without impurities (different onset temperature) and also differing by the equipment used, e.g., Differential Scanning Calorimetry (DSC) with continuous heating and cooling or Adiabatic Calorimetry (AC) without. These ways are often not used appropriately. For literature data, unless selected carefully, the uncertainty in Δ_pc_*H* is typically between 2% and 10%, that in *T*_pc_ is typically between 0.1 K and 2 K, thus, e.g., at 300 K between 0.03% and 0.7%. For example, for octadecane, Faden et al. [1] reviewed literature data and found values of the melting temperature between 298.36 K and about 303.65 K and for the melting enthalpy between 200 J/g and 256.7 J/g. Taking into account that the uncertainty in *T*_pc_ is very small, the uncertainty in Δ_pc_*S* (calculated via Equation (3)) is roughly as for Δ_pc_*H*, so between 2% and 10%.

### 2.2. Units and Notation

The units and notation used for the phase change enthalpy and temperature, in PCM R&D as well as other literature in Physics and Chemistry, can be confusing.

The units used, if looking at applications, are for the phase change enthalpy Δ_pc_*H* either volume-specific, e.g., in kJ/L, or mass-specific, e.g., in J/g, while for the phase change temperature *T*_pc_ the unit is always °C. For an understanding of the thermodynamics, it is more suitable for the phase change temperature *T*_pc_ to use Kelvin K and for the phase change enthalpy Δ_pc_*H* and entropy Δ_pc_*S* to use amount specific values, e.g., J/mol and J/(mol∙K).

Regarding notation, the International Union of Pure and Applied Chemistry (IUPAC) recommends [2] denoting enthalpy by *H* and entropy by *S*, their changes by Δ, and to use subscripts to Δ to denote a process, and to *H* and *S* to denote molar values by m. In this text, the process of phase change in general is denoted by pc. Then, for phase change in general and molar values, Equation (3) becomes

∆_pc_*H*_m_ = *T*_pc_∙Δ_pc_*S*_m_. (5)

While for the solid–liquid phase change, IUPAC recommends fus for fusion, and common literature also uses f, here, sl is used and complemented by ss for solid–solid phase change. In common literature, Δ is also often omitted and then, e.g., the subscript m in *H*_m_ could denote melting or molar value. Further on, *h* and *s* are also often used for specific values.

### 2.3. Data Basis and Sources

Thermodynamic data, specifically of the phase change enthalpy Δ_pc_*H* and phase change temperature *T*_pc_, are experimentally determined by calorimetric measurements and available for thousands of materials. Therefore, a huge data basis to also calculate the phase change entropy Δ_pc_*S* using Equation (3) already exists. General compilations are the CRC Handbook of Chemistry and Physics, Section 6, Enthalpy of Fusion [3], and the NIST Chemistry WebBook, Standard Reference Database [4]. Specifically for organic materials, extensive sources are the works by Acree and Chickos [5,6]. More compilations are available on specific material classes, e.g., for the homologous series of the n-alkanes. In some cases, the thermodynamics is also analyzed, even including entropy. The Results section summarizes the key findings from the reviewed literature.

## 3. Results

### 3.1. Graphical Analysis of H, T, and S Data for Trends and Correlations

Material property charts give a fast overview of values of many materials, useful for material selection. Materials from a material class, with similar particles and interactions, usually have similar properties and then form clusters. Even more, graphical analysis allows to identify trends in a series of materials and correlations between groups and is the easiest form of analysis to reveal possible common origins.

#### 3.1.1. The Elements

**Figure 1:**
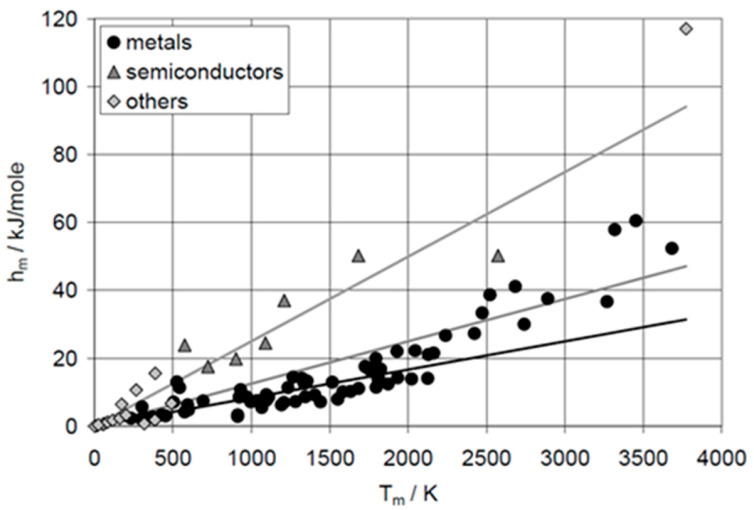
Molar melting enthalpy *h*_m_ versus melting temperature *T*_m_ of the elements with atomic number AN = 1 to 95. The lines indicate proportionalities with a factor 1, 1.5, and 3 (source: Mehling and Günther [7]).

Despite comprising only a few potential PCMs, e.g., Al and Cu, elements provide many significant insights. Already just plotting Δ_pc_*H* versus *T*_pc_, the two most application relevant properties, shows crucial things. For the solid–liquid phase change, meaning melting, there is an overall tendency of Δ_sl_*H* to rise with *T*_sl_. For the elements, this is shown in Figure 1. If the material class is specified, it reveals that material classes often have a correlation between Δ_sl_*H*_m_ and *T*_sl_, in Figure 1, e.g., a proportionality for metals and semiconductors.

The proportionalities between molar melting enthalpy Δ_sl_*H*_m_ and temperature *T*_sl_ were recognized early and rules stated to describe them. They have the general form (R = 8.31 J∙mol^−1^∙K^−1^ is the gas constant)

∆_sl_*H*_m_ = X∙R∙*T*_sl_. (6)

Alefeld [8,9] collected some of them. Well known is Richard’s rule (≈1880), which states that for metals X = 1 to 1.5. As Figure 1 shows, the rule describes many but not all metals. For semiconductors, X = 3 is stated. Again, Figure 1 shows that it describes many but not all. Comparing the general form of the rules, Equation (6), with the applicable form of Equation (3), which is

∆_sl_*H*_m_ = *T*_sl_∙Δ_sl_*S*_m_, (7)

shows that the proportionality values actually refer to the entropy change by

X = ∆_sl_*S*_m_/R. (8)

**Figure 2:**
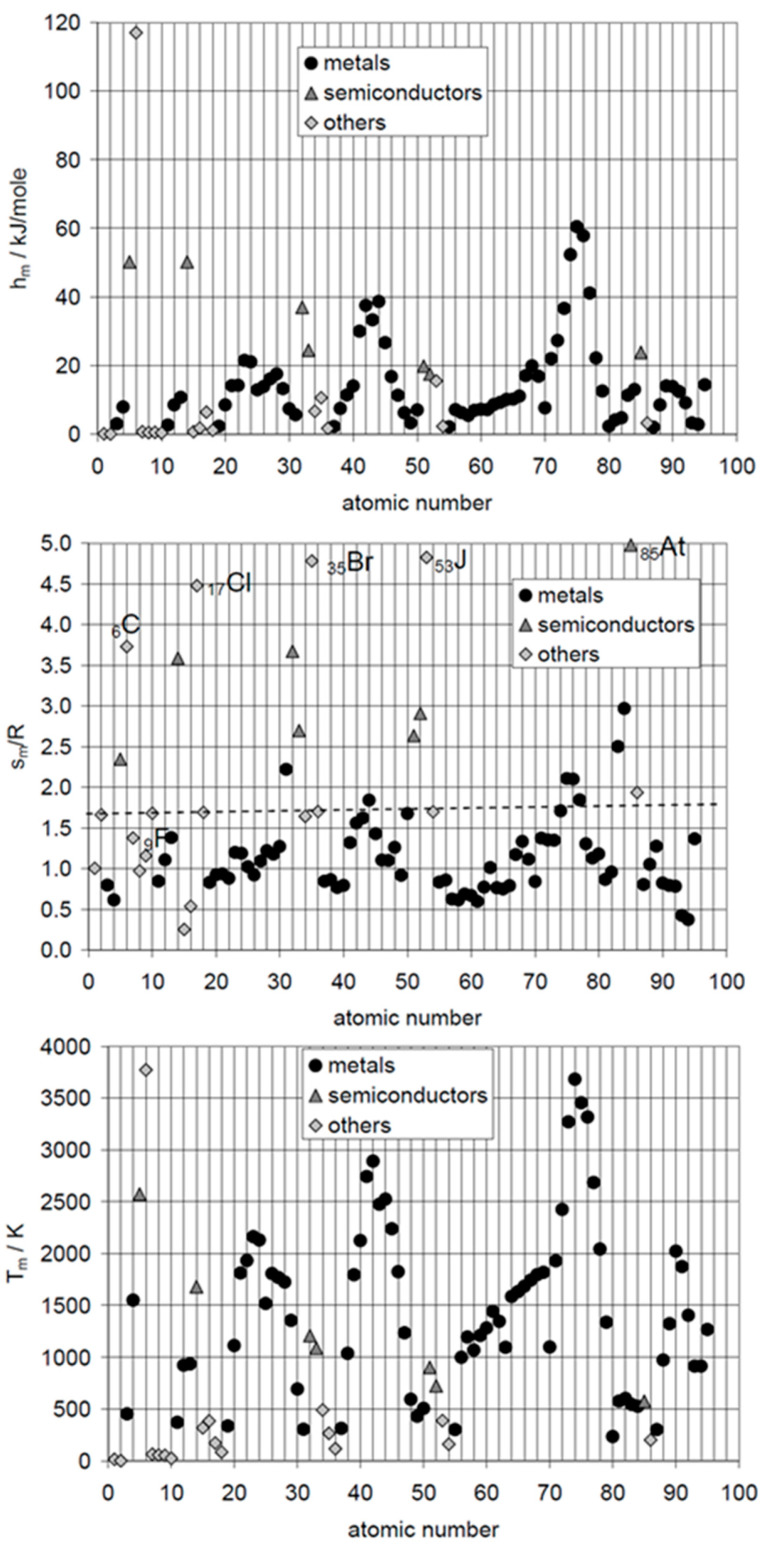
Top down: molar melting enthalpy *h*_m_, molar melting entropy *s*_m_, and melting temperature *T*_m_ of the elements versus atomic number AN = 1 to 95 (source: Mehling and Günther [7]).

**Figure 3:**
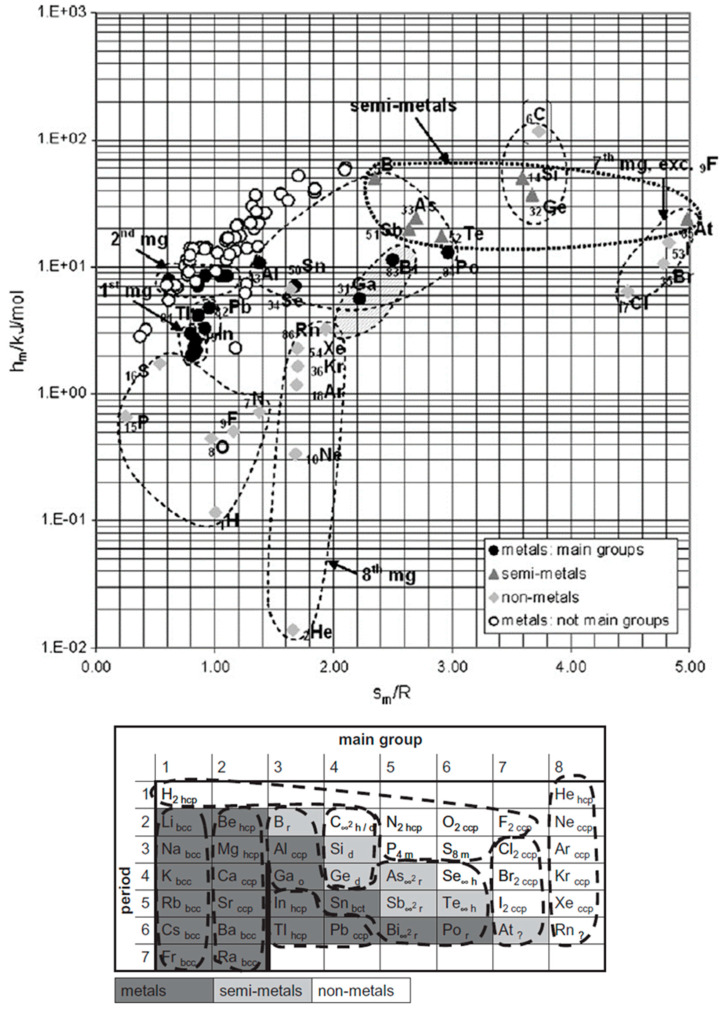
Molar melting enthalpy *h*_m_ versus entropy *s*_m_ divided by R for the elements with atomic number AN = 1 to 95; the identified elements with similar behavior are marked below in the PSE (source: Mehling [11]).

The logical next step for elements is plotting Δ_sl_*H*_m_, calculated Δ_sl_*S*_m_, and *T*_sl_, versus atomic number AN. De Podesta [10] did this for Δ_sl_*H*_m_ and *T*_sl_ but not for Δ_sl_*S*_m_. Mehling and Günther [7] included Δ_sl_*S*_m_, as shown in Figure 2. No general trend or correlation is seen. However, Δ_sl_*S*_m_ shows some interesting details. The noble gases _2_He, _10_Ne, _18_Ar, _36_Kr, _54_Xe, and _86_Rn have almost identical Δ_sl_*S*_m_, shown by a dashed line. Thus, they could be described well by an own rule. The halogens _17_Cl, _35_Br, _53_J, and _85_At, show very high and similar values, except the first, _9_F. The findings from the graphical analysis of Δ_sl_*S*_m_ versus AN on the noble gases and on the halogens led to a systematic test of options of graphical analysis. Plotting Δ_sl_*H*_m_ versus Δ_sl_*S*_m_ turned out to be fruitful, as Figure 3 shows.

Figure 3, showing Δ_sl_*H*_m_ versus Δ_sl_*S*_m_, [11] shows more clearly the difference between the noble gases, the halogens, and other elements, and that metals from main groups, those not from main groups, semi-metals (semiconductors), and others occupy distinct areas. These even fit to their location in the Periodic System of Elements (PSE). Contrary to the rules, many do not have similar Δ_sl_*S*_m_, but instead, Δ_sl_*S*_m_ varies correlated with Δ_sl_*H*_m_.

#### 3.1.2. The n-Alkanes

The n-alkanes, the most prominent homologous series of organic compounds, are analyzed quite often.

The correlation of Δ_pc_*H* with *T*_pc_, important regarding applications, seems to receive not much attention. The data are tabulated in many sources, but not a single source was found that investigated them graphically.

The correlation of *T*_pc_ with *n*, the number of carbon atoms, has been investigated by many researchers. Due to their importance, solid–liquid (sl) and solid–solid (ss) phase changes are investigated by many. Broadhurst [12] tabulated *T*_sl_ and *T*_ss_ of the n-alkanes with *n* between 1 and 100, and plotted them versus *n*, for odd and for even *n* in separate plots (*n* = 8 to 50). For odd and for even *n*, a general increase with *n* is observed, first sharp and later less. Dirand et al. [13] did the same for *n* = 8 to 45, with the same observation. For the lower *n* range, Boese et al. [14] investigated *T*_sl_ for *n* = 1 to 9, Costa et al. [15] for *n* = 1 to 16, and Dall’Acqua and Della Gatta [16] for *n* = 2 to 12, and plotted all *n* in a single plot as one curve. The same trend is observed. In addition, a systematic difference between odd and even *n* seems to exist. Soodoo et al. [17] plotted *n* = 1 to 46 with odd and even *n* as separate curves, which clearly shows a systematic difference.

The correlation of Δ_pc_*H* and of Δ_pc_*S* with *n* has also been investigated. In most sources, the difference between odd and even *n* is mentioned again. Soodoo et al. [17] plotted the mass-specific solid–liquid phase change enthalpy Δ_sl_*H* versus *n* for *n* = 8 to 30 in a single plot, with odd and even *n* as separate curves. For both a trend to rise with *n* and flatten out is observed, although somewhat different. Mass-specific and, in addition, molar values Δ_sl_*H*_m_ of *n* = 1 to 16 were plotted by Costa et al. [15]; they observed an almost linear rise, with systematic odd and even *n* deviations. Similar observations were also made by Dall’Acqua and Della Gatta [16] analyzing *n* = 2 to 12. Molar solid–liquid and solid–solid phase change enthalpies of the n-alkanes with *n* = 1 to 43, with odd and even *n* together, were plotted by Broadhurst [12]. A general increase with *n* for all phase changes was observed: for the solid–liquid phase change, linear, and for the solid–solid phase change, in some cases, non-linear.

**Figure 4:**
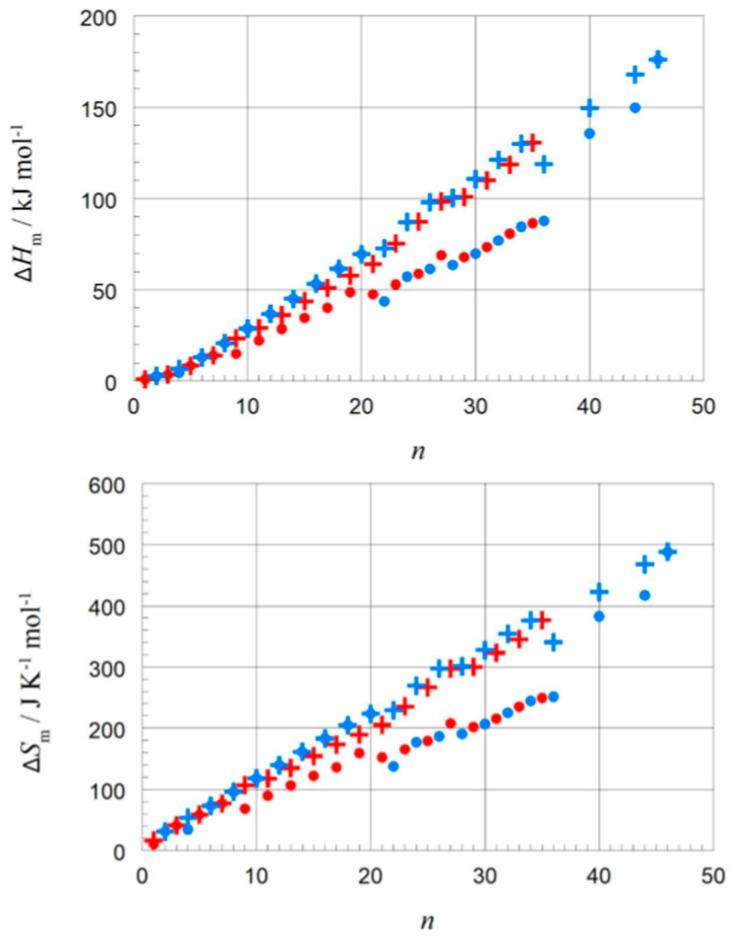
Molar change in enthalpy and of entropy versus *n* for n-alkanes; • for solid–liquid and + for total values (solid–liquid + solid–solid), with blue for even and red for odd *n* (source: Mehling and White [18]).

**Figure 5:**
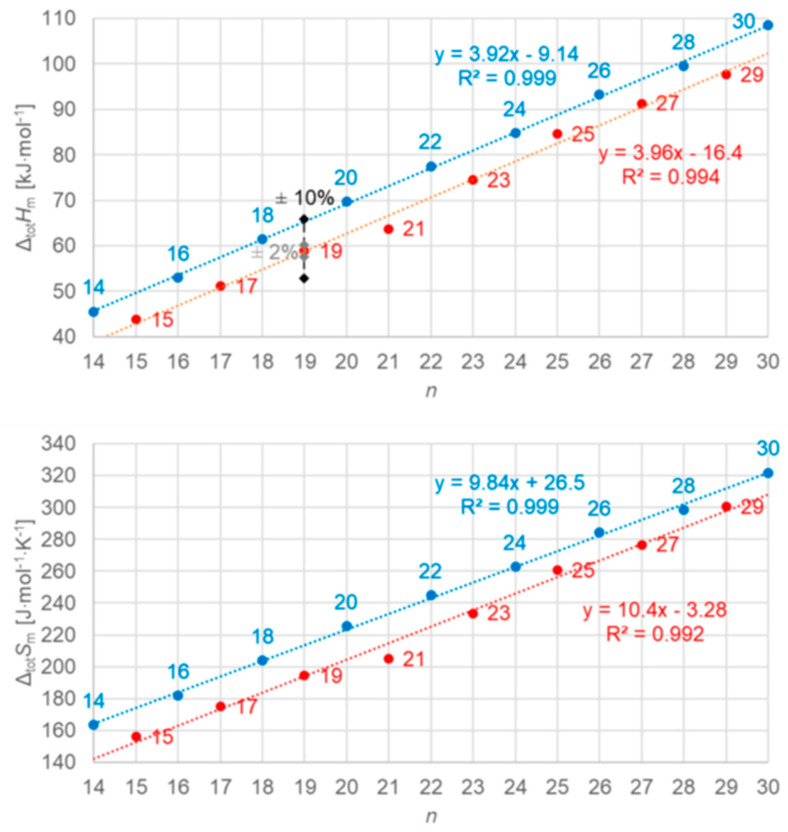
Molar total phase change enthalpy and entropy versus *n* for n-alkanes, with blue for even and red for odd *n* (source: Mehling et al. [19]). For enthalpy, the common uncertainties of data are included.

**Figure 6:**
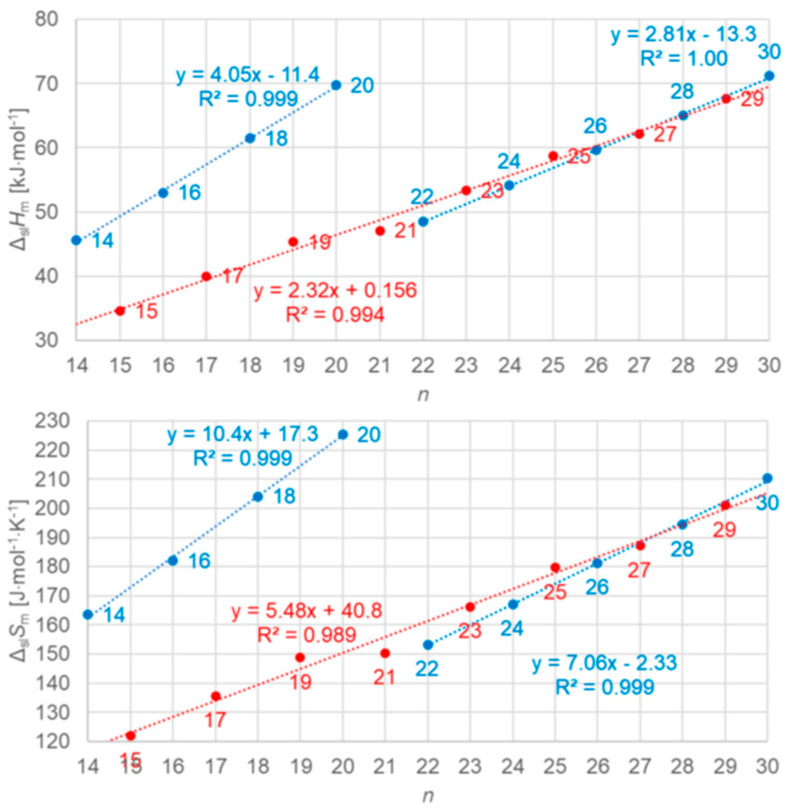
Molar solid–liquid phase change enthalpy and entropy versus *n* for n-alkanes, with blue for even and red for odd *n* (source: Mehling et al. [19]).

Dirand et al. [13] plotted for *n* = 1 to 44 the solid–liquid and, in addition, the total values (solid–liquid + solid–solid) and observed for both a linear increase with no visible odd and even *n* difference. Mehling and White [18] collected molar data covering *n* = 1 to 46 and plotted the same, shown in Figure 4. The data show a roughly linear rise except for small *n*, and systematic odd and even *n* difference in the total values, but also several outliers. As a consequence, Mehling et al. [19] performed a new analysis of n-alkane data, specifically from high-accuracy and high-resolution calorimetry, covering *n* = 14 to 30. For total values, shown in Figure 5, a perfect odd and even *n* difference is observed, each with its own linear correlation. For the solid–liquid phase change, shown in Figure 6, the behavior differs not by odd or even *n*. Instead, it is by the presence of a solid–solid phase transition or by its absence (the case for *n* = 14, 16, 18, 20). The molar melting entropy Δ_sl_*S*_m_ has also received some attention. Soodoo et al. [17] observed for *n* = 12 to 30 a trend to rise with *n*. Dall’Acqua and Della Gatta [16] observed for *n* = 2 to 12 a general trend to rise, with odd and even *n* deviations for *n* > 7. The data from Mehling and White [18] for *n* = 1 to 46, shown in Figure 4, show again a roughly linear rise for solid–liquid and also total values (solid–liquid + solid–solid), and a systematic odd and even *n* difference in the total values. The entropy data look somewhat alike the enthalpy data, again also with some outliers. Mehling et al. [19], using high-accuracy and high-resolution data for *n* = 14 to 30, confirmed the odd and even *n* difference for the total values, as shown in Figure 5, with own parallel linear correlations. For the solid–liquid phase change, shown in Figure 6, again, as for Δ_sl_*H*_m_, those without solid–solid transitions are different.

**Figure 7:**
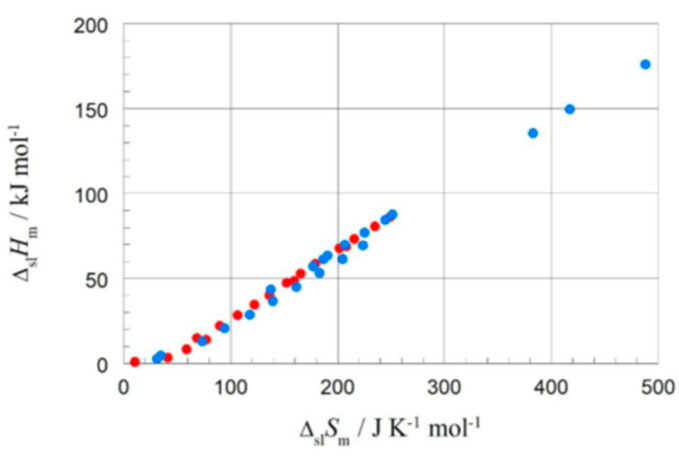
Molar solid–liquid phase change enthalpy versus entropy of alkanes, with blue for even and red for odd *n* (source: Mehling and White [18]).

The correlation of Δ_pc_*H* with Δ_pc_*S*, which gave valuable information for the elements, has up to now apparently only been investigated by very few researchers. Soodoo et al. [17] plotted the molar solid–liquid phase change enthalpy Δ_sl_*H*_m_ versus entropy Δ_sl_*S*_m_, with odd and even *n* marked separate, showing a roughly common linear correlation for larger *n*. Dall’Acqua and Della Gatta [16] observed for *n* = 2 to 12 separate ones. Mehling and White [18], as shown in Figure 7, observed also an overall linear trend, except for very small *n*. At a first glance, there also seems to be some odd and even *n* effect. Mehling et al. [19], analyzing high-accuracy and high-resolution data for *n* = 14 to 30, then revealed details.

**Figure 8:**
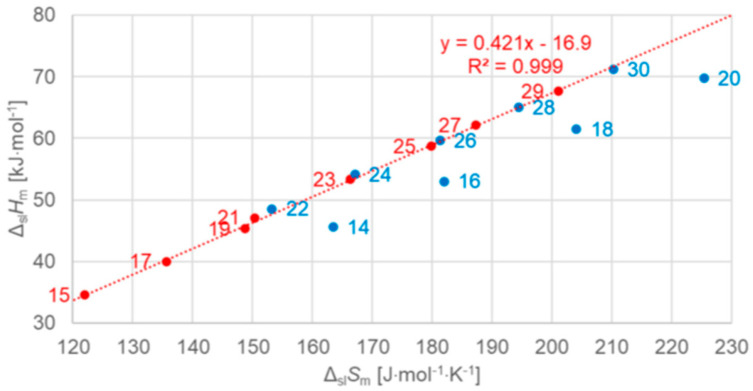
Molar solid–liquid phase change enthalpy versus entropy for n-alkanes, with blue for even and red for odd *n* (source: Mehling et al. [19]).

**Figure 9:**
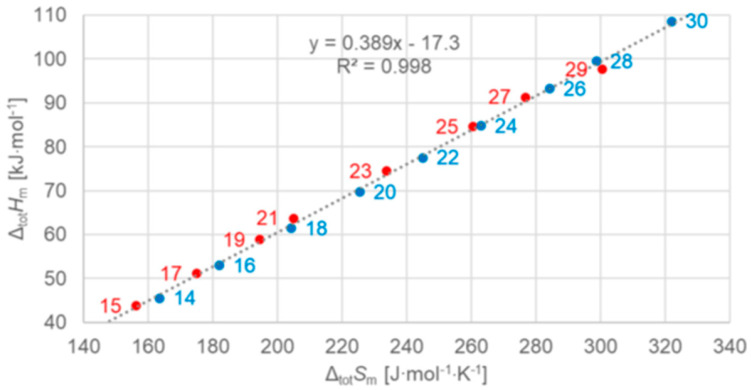
Molar total phase change enthalpy versus entropy for n-alkanes, with blue for even and red for odd *n* (source: Mehling et al. [19]).

As shown in Figure 8, different are again *n* = 14, 16, 18, and 20, which have no solid–solid phase transition, the same as was found analyzing the data set with regard to the correlations of Δ_sl_*H*_m_ and Δ_sl_*S*_m_ with *n* in Figure 6. However, although the plots of Δ_sl_*H*_m_ and Δ_sl_*S*_m_ versus *n* in Figure 6 look alike, there is an important additional detail. Figure 8 shows that all *n* with a solid–solid transition actually have the same Δ_sl_*H*_m_ versus Δ_sl_*S*_m_ correlation. For total values (solid–liquid + solid–solid), Mehling and White [18] found a linear correlation for *n* = 1 to 46. Mehling et al. [19], using high-accuracy and high-resolution data for *n* = 14 to 30, found that it is almost perfect, as Figure 9 shows.

#### 3.1.3. Other Homologous Series: The Alkanols, Carboxylic Acids, etc.

Aside from the n-alkanes, other homologous series of organic compounds were analyzed in the past. However, the individual ones do not add new key insights. Therefore, they are just briefly discussed, and the focus is on their main common features and crucial differences.

The correlation of Δ_pc_*H* with *T*_pc_, important regarding applications, seems not to receive much attention, as was already the case for the n-alkanes. Not a single source was found that has investigated it.

The correlation of *T*_pc_ with *n* is receiving more attention. Kahwaji and White [20] plotted the solid–liquid phase change temperature *T*_sl_ besides for n-alkanes also for n-alkanols (fatty alcohols), n-alkanoic acids (fatty acids, carboxylic acids), fatty esters, and others, in a single plot. It shows that all have the same tendency to rise with *n*, first steep and then, at large *n,* less steep. Some show an odd and even *n* variation. Their data covered *n* ≥ 8. Soodoo et al. [17] also plotted *T*_sl_ not only for alkanes but also other homologous series, in a single plot. For all they also observed the same trend to rise with *n*, less steep for large *n*. However, as their data cover *n* ≥ 1, they could observe that at small *n*, between *n* = 1 and 10, many show a decrease. Costa et al. [15] plotted *T*_sl_ besides for alkanes also for homologous α,ω-disubstituted alkanes, e.g., alkane-α,ω-diols, for *n* ≥ 1. They observed a trend to rise with *n* and an odd and even *n* difference, with two exceptions: alkane-α,ω-dioic acids and alkane-α,ω-diamides do not show a trend to rise. Dall’Acqua and Della Gatta [16] compared alkanes with alkane-α,ω-diamines for *n* = 2 to 12, and observed a trend to rise with *n* and an odd and even *n* variation.

The correlation of Δ_pc_*H* and of Δ_pc_*S* with *n* was investigated by most of those who also investigated *T*_pc_. Regarding Δ_pc_*H*, Kahwaji and White [20] plotted the mass-specific solid–liquid phase change enthalpy Δ_sl_*H* besides for alkanes also for fatty alcohols, observing a rise with *n* and, for fatty acids, observing the same together with some odd and even *n* variation. Soodoo et al. [17] also plotted mass-specific Δ_sl_*H* for alkanes and other homologous series and observed, for most of them, a trend to rise with *n*. Mass-specific and molar values Δ_sl_*H*_m_ (at 298 K) of *n* = 1 to a maximum of 16 were plotted by Costa et al. [15], besides for alkanes also for homologous α,ω-disubstituted alkanes, e.g., alkane-α,ω-diols. Generally, an odd and even *n* difference is observed. For mass-specific values, there is a trend to rise with *n* for some, while for others, there is not. In contrast, for molar values, all rise systematically with *n*. This shows the importance of looking at both of these values. Dall’Acqua and Della Gatta [16] investigated molar values of alkane-α,ω-diamines for *n* = 2 to 12 and observed also for them a trend to rise with *n* and an odd and even *n* variation. Mehling and White [18] plotted molar values for alkanes, alcohols, and fatty acids, and observed for all roughly linear trends to rise with *n*. Regarding Δ_pc_*S*, mass-specific and molar values Δ_sl_*S*_m_ (at 298 K) of *n* = 1 to a maximum of 16 were plotted by Costa et al. [15], besides for alkanes also for homologous α,ω-disubstituted alkanes. Generally, an odd and even *n* difference is observed. For mass-specific values, there is a trend to decrease with *n* for some of them, for some not. In contrast, for molar values all of them have a trend to rise with *n*. Mehling and White [18] plotted molar values for alkanes, alcohols, and fatty acids, and also observed a roughly linear trend to rise with *n* for all. Soodoo et al. [17] investigated also molar values and observed linear trends to rise with *n* for all and, specifically, separate linear and almost parallel correlations for monoesters, diesters, and diamides. Dall’Acqua and Della Gatta [16] investigated alkane-α,ω-diamines for *n* = 2 to 12 and observed the same trend to rise with *n*, and an odd and even *n* difference for *n* > 5.

**Figure 10:**
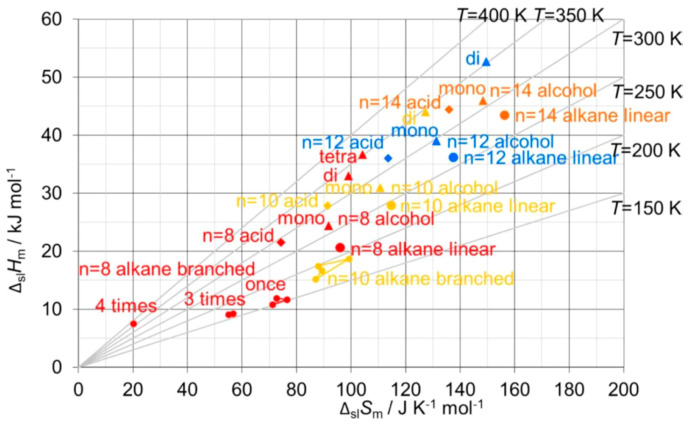
Molar solid–liquid phase change enthalpy versus entropy for linear alkanes (●), alcohols (▴), and fatty acids (◆), for *n* = 8 (red), 10 (yellow), 12 (blue), and 14 (orange), and, additionally, some branched alkanes and poly-alcohols (source: Mehling and White [18]).

The correlation of Δ_pc_*H*_m_ with Δ_pc_*S*_m_ has been apparently only investigated by very few researchers. For the solid–liquid phase change, Δ_sl_*H*_m_ versus Δ_sl_*S*_m_, Soodoo et al. [17] plotted not only values of alkanes, even and odd *n*, but also of fatty acids, fatty alcohols, mono- and diesters, and others in a single plot. It shows a linear correlation for all, most close together, except for diamides. Dall’Acqua and Della Gatta [16] plotted values of alkanes and alkane-α,ω-diamines, odd and even *n* separate for each, in a single plot. It shows an almost perfect linear correlation for all, with the fitted lines being parallel. Mehling and White [18] investigated besides alkanes also alcohols and fatty acids. Figure 10 shows, among other things, the data points for *n* = 8, 10, 12, and 14. Again, there is an almost perfect linear correlation for the data of the alkanes, the alcohols, and the fatty acids, and the lines are parallel. Additionally, for each individual *n* the data points of the alkane, alcohol, and fatty acid, differ by a similar shift.

#### 3.1.4. Other Compounds

While it can be suspected that more correlations can be found in a more comprehensive graphical analysis of elements and compounds in general, there is little systematic research in that direction. Mehling [21] investigated 1120 different materials (elements and chemical compounds), but not looking for trends and correlations. The focus was instead on the highest possible storage density. For this reason, the investigation did not use molar values but instead molar values divided by the number of atoms per mol. For example, the molar values of the halogens, being diatomic, were divided by 2. Plotting the corresponding enthalpy versus entropy data, no substance was found with a value of Δ_sl_*S*_m_/R per atom exceeding 4. About the highest values have the elements C, Si, and Ge, shown in Figure 3. Other “outliers” were, e.g., among the compounds with four to six atoms, FeCl_3_, AlCl_3_, VF_5_, and RuF_5_. They have high values of Δ_sl_*H*_m_ per atom (molar value divided by the number of atoms), with extraordinary high values of Δ_sl_*S*_m_ per atom and, thus, unusually low *T*_sl_. Despite not specifically looking for trends and correlations, some were still identified. For example, HCl, HBr, and HI have a trend of Δ_sl_*H*_m_ to rise while having similar Δ_sl_*S*_m_, and LiF, LiCl, LiBr, and LiI have a trend for both to decrease. LiF and LiCl have been listed as PCMs by Liu et al. [22]. Palomo Del Barrio et al. [23] investigated sugar alcohols and plotted *T*_sl_ versus *n*, Δ_sl_*S*_m_ versus *n*, as well as Δ_sl_*H*_m_ versus *T*_sl_, and compared different classes of sugar alcohols. No clear systematic trend or correlation was observed.

#### 3.1.5. Summary

Graphical analysis, by plotting Δ_pc_*H*_m_ versus *T*_pc_, by plotting *T*_pc_, Δ_pc_*H*_m_, and Δ_pc_*S*_m_ versus AN or *n*, and by plotting Δ_pc_*H*_m_ versus Δ_pc_*S*_m_, proved to be successful in identifying trends and correlations between materials. Regarding the goal to understand the thermodynamics of PCMs, trends and correlations in material properties indicate a possible common origin. Adding entropy to the analysis does not just add another set of plots. Entropy adds significant new information. This is especially the case plotting Δ_pc_*H*_m_ versus Δ_pc_*S*_m_, looking at the elements (Figure 2 compared to Figure 3), the homologous series of the alkanes (Figure 8 and Figure 9), and other homologous series (Figure 10). This is not surprising, as enthalpy and entropy relate to particles and interactions in materials in a different way. Using both as axes in a plot allows analyzing correlations and differences in the way they do this. It must be stressed that, to see details reliably, the accuracy and resolution found in common literature data is often not sufficient.

### 3.2. Analysis Using Information on Particles and Their Interactions

The next logical step towards understanding the thermodynamics of PCMs is to combine trends and correlations in material properties, identified by graphical analysis, with knowledge about the particles and their interactions. The correlations point to a probable common origin; thus, they are easily compared with knowledge on particles and interactions.

For the further analysis, it must be stressed that taking particles and their interactions as the origin of all material properties, all enthalpies, entropies, and changes Δ_pc_*H* and Δ_pc_*S* are direct consequences of them; however, all phase change temperatures *T*_pc_ are a consequence of the latter by Equation (3). This is illustrated in Figure 10, having Δ_sl_*H*_m_ and Δ_sl_*S*_m_ as its y- and x-axis and lines of constant *T*_sl_ calculated by Equation (3) included. This also explains why in the case of the elements Figure 3 with Δ_pc_*H* versus Δ_pc_*S* gives new insights, while Figure 1 with Δ_pc_*H* versus *T*_pc_ does not. A consequence of *T*_pc_ being only indirectly related to particles and their interactions is that trends in *T*_pc_ cannot be explained by them directly but only via the intermediate steps of Δ_pc_*H* and Δ_pc_*S*. Thus, the following discussion omits any further discussion of phase change temperature *T*_pc_ data.

#### 3.2.1. The Elements

The elements are composed of only one type of atom and thus are comparatively simple materials to study. Further on, the particles and interactions are well characterized and documented. An additional crucial advantage of elements is that most are monoatomic, which simplifies understanding greatly. The noble gases He, Ne, Ar, Kr, Xe, and Rn, in the eighth group of the PSE, are monoatomic. They have weak electromagnetic interaction due to induced dipoles, and their atoms can be considered spherical. This fits well to the observed properties in Figure 3, with almost the same Δ_sl_*S*_m_ and small Δ_sl_*H*_m_. The semimetals (semiconductors) C, Si, and Ge, in the fourth group of the PSE, are monoatomic and have covalent bonds in the solid and metallic bonds in the liquid. Crucial is that covalent bonds are directed; thus, they force neighbor atoms into a well-defined position, while metallic bonds are weaker and not directed. This also fits well to the observed properties in Figure 3, with large Δ_sl_*S*_m_ and also large Δ_sl_*H*_m_. In comparison, the metals are monoatomic and have metallic bonds in the solid and in the liquid. Compared to semimetals this should lead to a lower entropy change, which fits to observed Δ_sl_*S*_m_ and Δ_sl_*H*_m_. While metals of the main groups have similar Δ_sl_*S*_m_, this is not the case for those not of the main groups; the reason is probably a difference in the interaction. It is necessary to stress that for noble gases, metals, and semimetals, being monoatomic, their observed Δ_sl_*S*_m_ and Δ_sl_*H*_m_ are directly and solely based on the interactions between the particles. The strength of interactions determines Δ_sl_*H*_m_ and their directionality Δ_sl_*S*_m_. The halogens F, Cl, Br J, and At, in the seventh group of the PSE, are diatomic. They have weak electromagnetic interaction due to induced dipoles, however because of being diatomic with direction. This fits well to the observed properties, as plotted in Figure 3, with a trend in Δ_sl_*S*_m_ and also in Δ_sl_*H*_m_. Being diatomic, thus two atoms interacting, explains the comparatively high molar values here. Striking is that F is not in the observed trend but far away. The explanation becomes clear in the next step of analysis. Deviating most from the simple model of solid–liquid phase change, assuming the same particles and type of interaction in both phases and just different interparticle distances, are As, Sb, Bi, Te, and Se. As, Sb, and Bi have a layer structure in the solid phase, while the liquid phase consists of 4-atomic molecules. Se forms chainlike structures in the solid phase, and the liquid phase contains rings and chains of Se_5_, Se_6_, Se_7_, Se_8_, and other molecules. Te has a chainlike structure in the solid phase, and the liquid phase contains chains and probably rings. Thus, all of these consist of independent small molecules in the liquid and associate in the solid by new covalent bonds into chain or layer structures. Compared to C, Si, and Ge, which are in the solid bound by covalent bonds in all directions and monoatomic in the liquid, having chain or layer structures in the solid and molecules in the liquid should reduce Δ_sl_*S*_m_ and Δ_sl_*H*_m_; this fits well to their position in Figure 3. More details, a discussion of other elements, and the references for the information on the particles and interactions can be found in [11,21].

#### 3.2.2. The n-Alkanes

The n-alkanes have the common particle structure H(CH_2_)_n_H with *n* ≥ 1, and have a zig-zag geometry. Thus, depending on *n* being odd or even, the molecule ends are oriented the same or a different way. The particles (n-alkane molecules) are the same in all phases, and the same holds for the interactions. Linear trends with *n* are not surprising, because interactions are between close parts of neighbor particles and thus overall increase with (CH_2_)_n_. The deviation from linearity at small *n* is also not surprising as there the (CH_2_)_n_ part loses dominance compared to the ends. Thus, information on the particles and interactions can explain some observations discussed in Section 3.1, e.g., made by Boese et al. [14], Soodoo et al. [17], or Mehling and White [18]. It might also explain the odd and even *n* difference in total values (Figure 5). Then, however, two questions arise. First, why differ solid–liquid data versus *n* in a different way, between those with and without a solid–solid transition (Figure 6), and the same for Δ_sl_*H*_m_ versus Δ_sl_*S*_m_ (Figure 8)? And second, why do Δ_tot_*H*_m_ versus Δ_tot_*S*_m_ values show a common linear correlation for all *n* (Figure 9)?

#### 3.2.3. Other Homologous Series: The Alkanols, Carboxylic Acids, etc.

As discussed in the previous section, for many other homologous series similar observations were made as for the alkanes: a roughly linear rise of Δ_pc_*H*_m_ and Δ_pc_*S*_m_ with *n*, a linear correlation in Δ_pc_*H*_m_ versus Δ_pc_*S*_m_, and some odd and even *n* differences. The observed linear correlations were either almost the same or at least parallel. This fits well to the explanations discussed before for the alkanes, with the functional group causing the difference. How consistent these effects are can be seen in Figure 10: the data points for alkanes, alcohols, and fatty acids, with *n* = 8, 10, 12, and 14, can be connected by a line, and the data points for each *n* are shifted in a similar manner. However, many homologous series, e.g., alkanols and carboxylic acids, have like *n*-alkanes solid–solid transitions. Thus, as for n-alkanes, e.g., for solid–liquid data a different behavior of those with and without a solid–solid transition must be expected. Such a detailed analysis however seems to be missing up to now.

#### 3.2.4. Other Compounds

As pointed out in the previous section, while it can be suspected that more correlations can be found in a more comprehensive graphical analysis of compounds in general, probably even among the elements, there is today no systematic research in that direction. Only Mehling [21] investigated Δ_sl_*H*_m_ versus Δ_sl_*S*_m_ of 1120 different materials (elements and chemical compounds) and identified some interesting cases that give new insight. HCl, HBr, and HI have a trend of Δ_sl_*H*_m_ to rise, and similar Δ_sl_*S*_m_. They seem to crystallize as molecules having hydrogen bonds. The polarity of the hydrogen bonds should have a decreasing trend, leading however to the expectation of decreasing Δ_sl_*H*_m_. LiF, LiCl, LiBr, and LiI have a trend of Δ_sl_*H*_m_ and Δ_sl_*S*_m_ to decrease. They are salts. The trend of Δ_sl_*H*_m_ to decrease could be explained by the decreasing electronegativity of the halogens. The trend of Δ_sl_*S*_m_ to decrease does however not fit to the non-directionality of ionic interactions. The correct explanation might be a spatial effect due to the size of the ions that causes a trend to pin the smaller halogens to fixed locations between the Li ions. Again, solely information on particles and their interactions is not sufficient to explain all observations. Last, but not least, among the “outliers” identified were FeCl_3_, AlCl_3_, VF_5_, and RuF_5_, which have high values of Δ_sl_*H*_m_ per atom (molar value divided by the number of atoms) and extraordinary high values of Δ_sl_*S*_m_ per atom, thus comparatively low *T*_sl_. For AlCl_3_, the information collected in [21] is as follows. AlCl_3_ melts at 192 °C, however only at pressures above about 2 bar; below it sublimes. This melting temperature is typical for weak bonds between molecules, but there is a significant difference. AlCl_3_ has different structures depending on the temperature and the state: in the liquid it exists as dimer (AlCl_3_)_2_ interacting by weak bonds (Wells [24]; Holleman et al. [25]), while in the solid the bonding is in an AlCl_3_ layer structure (Wells [24]) with ionic bonds (Holleman et al. [25]). Thus, AlCl_3_ changes the bond type and also its molecular structure between solid and liquid. This also seems to be the case for the other “outliers” FeCl_3_, VF_5_, and RuF_5_.

Aside from the investigation of compounds in a general way, there is a specific issue of interest: isomers. Isomers are molecules with the same atomic composition, however different e.g., in molecular structure. Alkanes are defined as acyclic branched or unbranched hydrocarbons having the general formula C_n_H_2n+2_. Unbranched, thus straight-chain alkanes, are commonly called normal or short n-alkanes. Structural isomers of the n-alkanes are, e.g., their branched counterparts. Branched alkanes receive little attention in PCM R&D, as they have smaller mass and volume-specific Δ_sl_*H* than unbranched alkanes. Despite being of little interest for applications, they can still add information on thermodynamic origins. Mehling and White [18] tested Δ_sl_*H*_m_ versus Δ_sl_*S*_m_ for various *n* = 8 alkane structural isomers, specifically 1-, 3-, and 4-times branched isomers. As Figure 10 shows, a systematic trend to lower Δ_sl_*H*_m_ and lower Δ_sl_*S*_m_ is observed with each additional branching. This is consistent with the expectation that branching of an alkane reduces the options to interact with nearest neighbor molecules.

#### 3.2.5. Summary

The combination of knowledge about the particles and their interactions with trends and correlations in material properties, identified by graphical analysis, has shown to be very successful in different ways. In many cases explanations were identified for observed trends and correlations. Again, adding entropy to the analysis often did not only give additional but actually crucial information. Explanations, at least in part, for observed behavior were found for the noble gases, the semiconductors C, Si, and Ge, the group As, Sb, Bi, Te, and Se, the metals, and also the salts LiF, LiCl, LiBr, and LiI. For the n-alkanes and other homologous series, linear trends as well as odd and even *n* differences in total values can be explained. For branched isomers, not following a linear trend can be explained too. However, there are also still observations without explanation. The n-alkanes show Δ_sl_*H*_m_ and Δ_sl_*S*_m_ differences depending on whether they also have solid–solid transitions or not. Because solid–solid transitions also exist in other homologous series, like the alkanols and fatty acids, this is a wide-ranging open question. Further on, the observed trend in HCl, HBr, and HI is yet not explained, and the same holds for the fact that F does not follow the clear trend of the other halogens.

Additionally, there is a fundamental issue. The “outliers” FeCl_3_, AlCl_3_, VF_5_, and RuF_5_, have high values of Δ_sl_*S*_m_ per atom. AlCl_3_ changes the bond type, as well as its molecular structure between solid and liquid. This also seems to be the case for the other “outliers”. The same is the case for As, Sb, Bi, Te, and Se. For C, Si, and Ge, the change in bond type is even obviously necessary: covalent bonds in the solid cannot continue to exist in the liquid as covalent bonds between particles do not allow fluid motion. Thus, the simple model of solid–liquid phase change, assuming the same particles and type of interaction in both phases and just different interparticle distances, does not describe all materials.

### 3.3. Analysis Using Information on the Particle Arrangement

Material properties originate from the particles and their interactions, often directly, but also indirectly. For many monoatomic elements and compounds a reasonable explanation for the observed behavior, meaning trends and correlations, can be found from knowing the particles and the type of interactions, as shown before. However, in some cases this information is not sufficient. It can also not be sufficient to explain the behavior beyond trends and correlations, to derive absolute values of Δ_pc_*H*, Δ_pc_*S*, and *T*_pc_. For PCMs, by Equation (3), to maximize Δ_pc_*H* at given *T*_pc_ requires maximizing Δ_pc_*S*. Because the entropy *S* describes disorder, Δ_pc_*S* is tightly connected to the particle arrangement. In liquids, the particles are commonly in a disordered arrangement, while in solids the arrangement of particles can be disordered, then called an amorphous solid, as well as ordered, then called a crystalline solid. Therefore, to maximize the solid–liquid phase change entropy Δ_sl_*S* in PCMs, the solid phase should be crystalline. Solid–solid phase changes can be between two crystalline states or between a crystalline and an amorphous (disordered) state.

#### 3.3.1. The Elements

For the solid–liquid phase change in elements, information on the solid crystal structure was already included by Mehling [11], as shown in the PSE in Figure 3, but was not relevant in the analysis. This might be explained if different crystal structures do not lead to significant thermal effects compared to melting. The inverse conclusion, that the same crystal structures allow no different observations, seems wrong. F has the same crystal structure as all other halogens (Figure 3), but it behaves differently. According to information collected in [11], F_2_ molecules can still rotate in the solid, while the molecules of other halogens cannot. This rotation reduces the phase change enthalpy and entropy. Although F is not an option for PCMs, the degree of freedom to rotate has important implications. While for monoatomic substances only the location is relevant, diatomic molecules have an orientation. As the example shows, rotation perpendicular to the axis can have an effect (around the axis does not). In complex molecules, like branched alkanes, not only the whole molecule can rotate but also the branches around their connecting bond.

#### 3.3.2. The n-Alkanes

**Figure 11:**
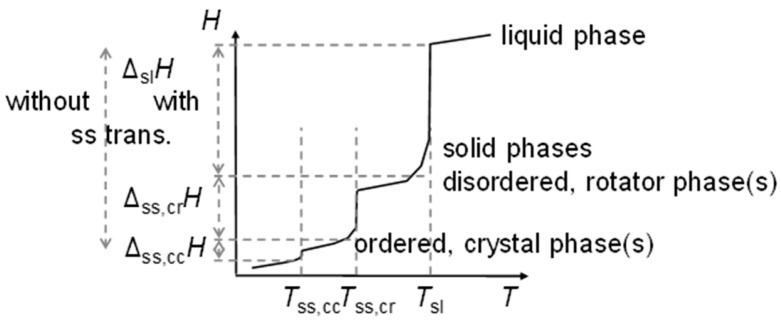
Schematic *H*(*T*) for n-alkanes, showing the different types of transitions.

The previous discussion (Section 3.1, Figure 6 and Figure 8, and Section 3.2) showed that while linear trends can be explained by *n*, the widespread statement that odd and even *n* behave different is not accurate. Precisely, odd and even *n* make a difference in the correlation of total values Δ_tot_*H*_m_ and Δ_tot_*S*_m_ with *n* (Figure 4 and Figure 5), but not in the correlation of Δ_tot_*H*_m_ with Δ_tot_*S*_m_ (Figure 9). For solid–liquid phase change, the presence or absence of solid–solid transitions makes the difference in the correlation of Δ_sl_*H*_m_ and of Δ_sl_*S*_m_ with *n* (Figure 6), and also in the correlation of Δ_sl_*H*_m_ with Δ_sl_*S*_m_ (Figure 8). Dirand et al. [13] offer an explanation. The n-alkanes have two types of polymorphous solid phases: ordered at low temperature, called crystal phases, and disordered at higher temperature, denoted rotator phases, as Figure 11 shows. Corresponding crystal–crystal (cc) transitions have small thermal effects, while crystal–rotator (cr) transitions have larger thermal effects and occur just below the melting temperature [13]. The presence or absence of them is crucial.

Dirand et al. [13] already stated that the enthalpy difference between the ordered phase and the liquid phase varies linearly as a function of *n* whatever its parity. This corresponds to Δ_sl_*H*_m_ without ss transition in Figure 11, meaning total values, and would be explained if all n-alkanes change from a comparable ordered solid state to the same liquid. However, Dirand et al. [13] did not see an odd and even *n* effect, which in reality exists (Figure 5). An explanation for the odd and even *n* difference in total values (lowest temperature crystal state to the melting) comes from the zig-zag form of the n-alkanes and different lowest temperature crystal structures. Broadhurst [12] and Boese et al. [14] provide graphical visualizations of the different arrangement of the molecules. For *n* = 14 to 30, all odd *n* have an orthorhombic crystal structure, while even *n* are triclinic except *n* = 28 and 30, which are monoclinic [13]. So, the crystal structure is probably the explanation. That the effect is absent in the correlation of total values Δ_tot_*H*_m_ with Δ_tot_*S*_m_ (Figure 9) implies a general enthalpy–entropy correlation. However, the full explanation must be more complex as the solid–liquid values do not show it (Figure 8). Dirand et al. [13] also stated that the melting enthalpy varies linearly as a function of *n* whatever its parity when fusion corresponds to a change of a disordered phase to the liquid. This corresponds to Δ_sl_*H*_m_ with ss transition in Figure 11, precisely with a crystal–rotator (cr) transition. This is the case for all, except for *n* = 1 to 8, 10, 12, 14, 16, 18, 20 [13]. The data from Mehling and White [18], in Figure 4, show the same observation. Consequently also, the solid–liquid data points for *n* without a crystal–rotator (cr) transition (*n* = 1 to 8, 10, 12, 14, 16, 18, 20) overlap with the data points of the total values. The high-accuracy and high-resolution calorimetry data by Mehling et al. [19] show that in detail (Figure 6): *n* = 14, 16, 18, and 20 have no solid–solid transition, thus they are melting directly from the lowest temperature crystal structure, and as expected, they have a larger value of Δ_sl_*H*_m_. For the phase change entropy, total as well as melting, the identified processes can be expected to lead to a similar behavior as for the enthalpies. This is the case in Figure 4, Figure 5 and Figure 6. Consequently, *S*(*T*) should behave similarly to *H*(*T*) shown in Figure 11. However, as the differences in Figure 8 show (*n* = 14, 16, 18, 20), only similar but not identical.

#### 3.3.3. Other Homologous Series: The Alkanols, Carboxylic Acids, etc.

The previously found explanations for certain behaviors of n-alkanes can also be expected to hold for fatty alcohols as well as for fatty acids. According to Mehling and White [18], solid–solid transitions exist in fatty alcohols for all investigated *n* with *n* ≥ 13, and for fatty acids for *n* = 7, 9, 11, 13, 15, 17, 19, 20, 21, 22, etc. This fits perfectly to the trends and correlations observed for Δ_tot_*H*_m_ and Δ_sl_*H*_m_ versus *n*, respectively Δ_tot_*S*_m_ and Δ_sl_*S*_m_ versus *n*, in the corresponding plots (corresponding to Figure 4 but not shown here). For the fatty alcohols the total values seem to indicate that there are also different lowest temperature crystal structures. Crystallographic data should be used in the future for verification.

#### 3.3.4. Other Compounds

Among the pure substances used as PCMs, water is most relevant. Water has different crystal structures, however not at ambient pressure. Upon cooling, if crystallization does not occur water becomes an amorphous solid, often called glass. This leads to a much lower solid–liquid phase change enthalpy. Using entropy for an analysis could be very interesting, especially as the same effect is also very common in eutectic water–salt mixtures used as PCMs for temperatures well below 0 °C. However, compared to the previous discussion, the glass state is a non-equilibrium state. That means the entropy cannot be calculated via Equation (3) anymore, as it holds only for thermodynamic equilibrium conditions. Other compounds that are of high interest are, e.g., sugar alcohols and polymers. At the moment, however, no investigation using entropy in the analysis seems to exist.

#### 3.3.5. Summary

Information on the particle arrangement, specifically whether the particles in a solid are in an ordered or in a disordered state and which one, can be helpful in understanding the thermodynamics of phase changes. For the n-alkanes, the crystal structure of the lowest temperature crystal, related to the zig-zag molecular structure with the odd and even *n* difference at the molecule ends, allows to understand the differences between odd and even *n* in total values (Figure 5). In addition, the presence or absence of transitions between different ordered or disordered states allows to understand the differences in the solid–liquid values. This holds for enthalpy and entropy alike. Similar observations must be expected for other homologous series.

For the monoatomic elements, enthalpy–entropy correlations were easy to explain already by the type of interaction (e.g., metallic, covalent etc.), if the type was changing between solid and liquid (e.g., C, Si, Ge) then taking both phases into account. That enthalpy–entropy correlations still exist for molecules with many interactions with neighbors, e.g., the n-alkanes, even with functional groups and, thus, different types of interactions, e.g., the fatty acids, and even for several transitions together in total values, is an outcome that is not straightforward and should be investigated further by more data analysis.

### 3.4. Calculation of Properties from Atomic and Molecular Interactions

The previous section has shown that, at some point, it becomes difficult to find explanations for observed trends and correlations. Even more, that an explanation fits to observations does not prove it is correct. At this point, a completely different approach than the ones used in the previous sections is promising. Since all properties are due to the particles and their interactions, they can be used as the starting point. While not new at all, this approach has seen an incredible development in recent years.

According to a review of models for the melting of crystalline solids by Mei and Lu [26], already in 1891 Sutherland developed a kinetic theory of solids. He considered atoms as hard spheres, which are vibrating, and assumed that melting occurs once their distances get beyond a certain value given relative to the atomic diameter. Simply put, a solid is treated as a package of spheres, and melting occurs if spheres can slide past each other. Calculations based on that model showed that the ratio of vibration amplitude to the atomic spacing is almost the same for all elements at their melting temperature. This is not surprising as most elements are monoatomic and can be considered as spheres. Noteworthy is that in all the reviewed models for melting the use of entropy is not mentioned (except to treat superheating). At the time of that review, 2007, computer modeling of particles and their interactions started to develop. Nowadays, advanced computing capabilities and more detailed knowledge of particles and interactions is available. As a result, computer modeling of particles and their interactions, called Molecular Dynamic Simulation (MDS), is used to study phase change as well as many other processes.

In MDS, commonly, the interaction of atoms and molecules is described by forces, and in successive time steps the equations of motion are used to calculate the motion, new position, and thus the development with time. MDS is, by its principle, able to simulate equilibrium states as well as non-equilibrium processes. Thus, MDS can even investigate processes starting in one phase only and ending in another, including supercooling, nucleation, and crystal growth. Being based on atoms and molecules in all detail, it can also treat complex molecules. However, MDS also has significant and fundamental limits, at least for now. First of all, modeling of interactions, e.g., with quantum mechanic effects, is not straight forward. The choice of appropriate potentials is complex and puts a first limit on the accuracy of MDS results. As in the real world, results also depend on chosen boundary conditions. Other issues, specifically with regard to PCMs, are the control and determination of temperature, enthalpy, and entropy. Macroscopic properties are, e.g., calculated from time-averaged ensembles. With regard to PCMs and the scope of this work, most crucial is the accuracy of the simulation results for *T*_pc_ and Δ_pc_*H*.

#### 3.4.1. The Elements

MDS of elements, specifically metals, has been conducted in the past by numerous researchers. For example, Belonoshko et al. [27] conducted an MDS study of iron melting, specifically for the high pressures in the earth interior. Kien and Trang [28] simulated structure transitions of amorphous and liquid aluminum. The literature review resulted however in little research on equilibrium phase change properties, and entropy was not mentioned at all in the studies.

#### 3.4.2. The n-Alkanes

MDS of n-alkanes has been performed by various researchers, and with focus on very different issues. Tafelmeier and Hiebler [29] tested MDS in order to simulate the crystallization of octadecane, *n* = 18, on a nucleating agent. For that, they also simulated the density, phase change temperature, and enthalpy, as well as the crystal structure. The enthalpy difference between 10 K below and above the melting point from simulation was about 10% higher compared to the experiment, the melting temperature about 1 °C lower. Burrows et al. [30] attempted a benchmarking of various Molecular Dynamics force fields for solid–liquid and solid–solid phase transitions in alkanes. They performed simulations including rotator phases and calculated density and melting temperature. Using different MDS models they found for the melting temperature of *n* = 15 values from 286 K to 317 K; the experimental value was 283 K, thus, the simulated values deviated by up to 12%. For *n* = 16 the results were from 274 K to 324 K; the experimental value is 291 K, thus simulated values deviated by up to 11%. They also calculated the entropy from probability distributions, however only to show its change with time in simulation runs. Burrows et al., in a later study [31], investigated the structure of the hexadecane rotator phase by combining X-ray spectra and MDS. The entropy was calculated, precisely the rotational entropy, and used qualitatively in the analysis. Iliev et al. [32] performed another study on the freezing of hexadecane by equilibrium molecular dynamic simulations, looking into details of rotator states, but also without discussing entropy.

#### 3.4.3. Other Homologous Series: The Alkanols, Carboxylic Acids, etc.

Regarding other homologous series, no MDS studies were found that treated phase change enthalpy or entropy.

#### 3.4.4. Other Compounds

Cheng et al. [33] used MDS to study phase change properties and underlying mechanisms for sugar alcohols, specifically focusing on melting and the associated latent heat. The sugar alcohols they investigated were glycerol, erythritol, arabinitol, and mannitol. The focus was on the different types of interactions and their contributions to the overall behavior. While for three sugar alcohols simulated and experimental melting enthalpies agreed perfectly (330 vs. 334 kJ/kg, 328 vs. 326 kJ/kg) or at least well (174 vs. 227 kJ/kg), for one, glycerol, it did not agree at all (52 vs. 200 kJ/kg). The authors attribute the disagreement to problems with the model for the interaction. However, glycerol is known for not crystallizing well thus that the experimental value should be taken with much care. Polyethylene (PE), a polymer, is among the early substances to be suggested as a PCM. Specifically when cross-linked it does not flow macroscopically anymore, which makes its application for heat storage much easier. Compared to most elements and many other substances with simple molecular structure it is however known to form crystals even from different parts of the same molecule. Thus, MDS is a promising approach for a better understanding of its thermodynamics. Lv and Ruan [34] used MDS to investigate the nonisothermal crystallization of a single PE chain and also of short chains. They investigated the effect of parameters like the cooling rate, PE chain length, etc., on crystallization, however, they did not look at the equilibrium phase change enthalpy, temperature, or entropy. Paajanen et al. [35] investigated the crystallization of cross-linked PE by MDS and focused on the effect of cross-link density. The results confirm some already expected effects, e.g., that an increase in cross-link density causes crystallization to decrease, and that cross-links are rejected into the amorphous inter-crystalline phase. As it was not their focus, their study also contains no results on equilibrium phase change enthalpy, temperature, or entropy.

#### 3.4.5. Summary

MDS, being comparatively new, has in the past years increasingly been used to study phase changes. Studies cover solid–solid and solid–liquid phase change, specifically nucleation and crystal growth, and to a lesser degree also equilibrium phase change enthalpy and temperature.

Among pure substances investigated, specifically with potential use as PCM, are metals, n-alkanes, water, sugar alcohols, polyethylene, and x-linked polyethylene. MDS has already given significant insight into many processes on the atomic and molecular level, e.g., nucleation, crystallization, and phase transitions between polymorphs (crystal and rotator phases). With regard to the most crucial properties for PCMs, the equilibrium phase change temperature and enthalpy, values still vary significantly with regard to closeness to experimental values. In some cases, the results from MDS are as close as high-quality calorimetric data, in others as far away as low-quality literature data. Entropy was only used in few investigations, then calculated from probability distributions and not via Equation (3), and not analyzed.

## 4. Discussion

In this review, the potential of entropy to contribute to an understanding of the thermodynamics of PCMs and the current state and use, are analyzed. The focus is on the enthalpy change on phase change Δ_pc_*H* and the phase change temperature *T*_pc_, and the scope is pure substances, thus elements and chemical compounds. Therefore, the following discussion is split into two parts. The first part discusses the findings directly related to the different tools of analysis. Key points are the already achieved understanding of the thermodynamics of PCMs, the role of entropy in the analysis, and lessons learned for any future analysis. The second part discusses briefly some far reaching findings, beyond the analysis tools, reaching further than the thermodynamics of PCMs.

### 4.1. Discussion of the Analysis Tools and the PCM-Related Findings

#### 4.1.1. Graphical Analysis of *H*, *T*, and *S* Data for Trends and Correlations

Graphical analysis by plotting Δ_pc_*H*_m_ versus *T*_pc_, by plotting *T*_pc_, Δ_pc_*H*_m_, and Δ_pc_*S*_m_ versus AN or *n*, and by plotting Δ_pc_*H*_m_ versus Δ_pc_*S*_m_, proved to be successful in identifying trends and correlations between materials. With regard to understanding the thermodynamics of PCM, trends and correlations in material properties indicate a possible common origin. For this, it is essential to use molar values.

In older literature, rules like Richard’s rule can be found which essentially assume a proportionality between Δ_pc_*H*_m_ and *T*_pc_, thus due to Equation (3) a typical Δ_pc_*S*_m_. R&D in the past decade using plots of Δ_pc_*H*_m_ versus Δ_pc_*S*_m_ showed, however, that there is instead a wide variety of material class typical enthalpy–entropy correlations. Individual correlations were identified for the elements (metals of main groups, metals not of main groups, halogens, noble gases, etc.), compounds in general, and for the homologous series of the n-alkanes and others, regarding solid–liquid as well as solid–solid transitions. In the latter case, to see the details, calorimetric data with sufficient resolution and accuracy were required. Further on, for a complete picture, looking at the different types of transitions, solid–liquid as well as solid–solid, and separate analysis as well as analysis of total values was necessary. This is probably needed for all homologous series.

For completeness, and because it is needed also later, it should be mentioned that a similar analysis of the liquid–gas phase change [36] also identified trends and enthalpy-entropy correlations beyond Trouton’s well-known rule.

Regarding the use of entropy in the analysis, it might surprise at a first glance that it leads to new insights as it is just calculated from the phase change enthalpy and temperature, the data used in analysis otherwise. However, what is crucial is the not information contained, but instead the information made visible. Because enthalpy and entropy relate to particles and interactions in materials in a direct and also different way, using both as the axes in a plot allows to reveal and analyze correlations and differences in the way they do this.

#### 4.1.2. Analysis Using Information on Particles and Their Interactions

The next logical step towards understanding the thermodynamics of PCMs is to combine trends and correlations in material properties, identified by graphical analysis, with knowledge about the particles and their interactions. Often this knowledge allows to understand already some of the trends and correlations observed, and again entropy plays a crucial role.

In many cases, for observed trends and correlations explanations were identified. Examples among the elements are the noble gases, the semiconductors C, Si, and Ge, the group As, Sb, Bi, Te, and Se, and the metals. Examples among the compounds are the salts LiF, LiCl, LiBr, and LiI, the n-alkanes, and other homologous series. For the latter, linear trends as well as odd and even *n* differences in total values can be explained.

A key finding using entropy in the analysis is that the simple model of phase change, having the same particles and the same interactions in the phases taking part in phase change, does not always hold. For C, Si, and Ge this is obvious. They have covalent bonds in the solid, which is not compatible with liquid flow. As liquids they are actually metallic. Other examples are As, Sb, Bi, Te, and Se, which also change their particle structure, thus undergo a chemical reaction. Another example is AlCl_3_, and maybe also the other “outliers” FeCl_3_, VF_5_, and RuF_5_, but for the latter the information is not fully clear. As a consequence, for future R&D, it is essential to have information on the particles and their interactions in both phases. Even more, these cases show unexpected high Δ_sl_*S*_m_, thus give a new direction to look for PCMs.

#### 4.1.3. Analysis Using Information on the Particle Arrangement

Although for many observations information on particles and their interactions is already sufficient to derive an explanation, there are exceptions. Here information on the particle arrangement is useful. As entropy describes disorder, it is specifically correlated with ordered or disordered solid phases.

For the n-alkanes, as example, the crystal structure of the lowest temperature crystal, related to the zig-zag molecular structure with the odd and even *n* difference at the molecule ends, allows to understand the differences between odd and even *n* in total values. In addition, the presence or absence of transitions between different ordered or disordered states allows to understand exceptions in the solid–liquid values. Similar observations must be expected for other homologous series, however, these have not been studied yet. As a consequence, for future R&D it is essential to have also information on the particle arrangement in the different phases.

#### 4.1.4. Calculation of Properties from Atomic and Molecular Interaction

MDS has been used to study solid–solid and solid–liquid phase change, e.g., regarding nucleation and crystal growth, and also for equilibrium phase change enthalpy Δ_pc_*H* and temperature *T*_pc_. With regard to the latter, the most crucial properties for PCMs, the closeness between simulated and experimental values varies a lot. Some results are as close as high-quality calorimetric data, others as far away as low-quality literature data. Regarding entropy, calculated Δ_pc_*S* via Equation (3) are only as accurate as Δ_pc_*H* and *T*_pc_ input from MDS. Moreover, entropy is only mentioned in very few investigations, and not analyzed at all. This raises the question of the importance and potential use of entropy in MDS. Actually, how is it possible that in some works simulated *T*_pc_ and Δ_pc_*H* values are quite accurate, despite not using entropy? The explanation is that because particles and their interactions determine all material properties, their simulation in MDS inherently also simulates entropy and its changes. Thus, even if entropy is not explicitly considered, MDS is inherently taking order and disorder into account, and if the simulated *T*_pc_ and Δ_pc_*H* values are quite accurate then also the entropy Δ_pc_*S* values are simulated accurately. Thus, even if not considered explicitly, entropy is inherently simulated, and thus, MDS results can and should be used to discuss order and disorder. Already today, MDS has led to significant insight into the thermodynamics of PCMs, e.g., regarding nucleation, crystallization, and phase transitions between polymorphs (crystal and rotator phases), thus non-equilibrium processes. Thus, MDS is not only adding information on the atomic and molecular level to the information gained by the other tools, but moreover additionally giving complementary information on non-equilibrium processes. For the use of entropy in MDS this means that the potential is even larger than when using equilibrium thermal data. The potential is however virtually untapped today.

### 4.2. Discussion of Far-Reaching Findings

Aside from analyzing the potential of entropy to contribute to an understanding of the thermodynamics of PCMs and the current state and use, some findings in past R&D are far reaching, beyond that scope.

#### 4.2.1. Model of Solid–Liquid and Solid–Solid Phase Change

The common literature assumes for the solid-liquid, as well as solid-solid, phase change that the same particles and type of interaction are present in both phases. However, the discussed results clearly show that this is not always the case. Upon solid–liquid phase change, C, Si, and Ge must change the type of interaction as covalent bonds between particles cannot exist in the liquid. For As, Sb, Bi, Se, and Te, different molecular structures in the solid and liquid phases exist. For the liquid–gas phase change such cases are well known (Mehling [36]), e.g., ZnTe, CdSe, and CdTe change phase between liquid (l) and gas (g) as MX(l) ⇋ M(g) + 0.5X_2_(g). A problem with these findings is, however, that literature often just states a particle structure and type of interaction in the liquid or solid, without clearly stating a change at the phase change temperature. This is different for the results of some recent R&D by Saher et al. [37] concerning a mixture, and thus not discussed in the results. They investigated the eutectic mixture of boric acid (H_3_BO_3_) and succinic acid ((CH_2_)_2_(CO_2_H)_2_), and determined a phase transition at around 150 °C with an extraordinary phase change enthalpy of 394 J/g. What is crucial is that they investigated the chemical process upon phase change in detail and proved that a chemical reaction takes place. The phase transition involves melting of the boric acid, its dehydration into metaboric acid and water (H_3_BO_3_ → HBO_2_ +H_2_O) and the water then being dissolved in the liquid. The water is then kept there until the transition is reversed on cooling, resulting in rehydration. Thus, the simple model of phase change, having the same particles with the same interactions in the involved phases, covers most materials, however not all; and the cases missed can show interesting properties. This also means that the classification of TES options, discussed in the Introduction, covers most materials but is not comprehensive.

#### 4.2.2. Enthalpy–Entropy Correlations

Aside from the enthalpy–entropy correlations discussed for solid–liquid and solid–solid phase change, and others briefly mentioned for liquid–gas phase change, more correlations exist in many areas of Chemistry. The analysis of PCMs might add new insights here, as with PCMs, the situation is often simpler. For the monoatomic elements, enthalpy–entropy correlations can be directly related to the properties of the different bond types affected upon phase change, simply because there is no other possible contribution. That enthalpy–entropy correlations still exist for molecules with many interactions with neighbors, e.g., the n-alkanes, even with functional groups and thus different types of interactions, e.g., the fatty acids, and even for several transitions together in total values, might be understood by superposition of several different enthalpy–entropy correlations. However, if and how far that holds is up to future R&D.

## 5. Conclusions—Future R&D Directions

The review showed that the use of entropy in the analysis can significantly contribute to an understanding of the thermodynamics of PCMs, specifically of the enthalpy change on phase change Δ_pc_*H* and the phase change temperature *T*_pc_. The review covered past R&D on pure substances, thus elements and compounds. For compounds, the focus was on n-alkanes and other homologous series.

Based on the results and the previous discussion, future R&D directions with respect to the thermodynamics of PCMs can be divided into four main topics.

### 5.1. Analysis Using Material Data

The first part covers analysis of material data, as discussed in the Results Section 3.1, Section 3.2, Section 3.3. Specifically, future R&D should deepen and extend the available data basis, and afterwards analyze it in the described ways. To deepen and extend the available data basis regarding Section 3.1, R&D could in the future also comprise the determination of whole *H*(*T*) curves from calorimetric measurements, at best on heating and on cooling. That way the data sets include also supercooling and nucleation. The data basis thus also becomes more complete with regard to comparison with the results of MDS studies. For Section 3.2, future R&D should keep in mind that particles and interactions can be different in different phases, so should be determined if there are doubts about them. For Section 3.3, future R&D should take into account solid–solid transitions as they can play a crucial role, and then include the crystal structures. Alkanes, and other homologous series, should be covered and analyzed starting *n* = 1, and later sugar alcohols etc. Last, but not least, despite that only a few are PCMs, the elements should also be analyzed in more detail; specifically monoatomic elements promise detailed information on interactions, useful in many areas.

### 5.2. Analysis by Simulating Particles and Their Interactions

The second part covers simulation, as discussed in the Results Section 3.4. Here a first step could be to test and improve MDS models by looking at the elements, specifically regarding modeling interactions. In a second step, simulation of whole *H*(*T*) curves could be conducted to see in more detail where deviations between simulated and experimental values occur, and in turn, possibly improve the models. This could start with equilibrium *H*(*T*) curves, e.g., from slow heating and cooling, and then be repeated for crystallization after supercooling. Once the accuracy and reliability of MDS regarding qualitative as well as quantitative results is established, MDS can unfold the full potential in PCM R&D.

### 5.3. Enthalpy–Entropy Correlation

In the Results section, a range of enthalpy–entropy correlations for solid–liquid and solid–solid phase change were identified. A general understanding of enthalpy–entropy correlations is of far-reaching importance as they are also found in liquid–gas phase changes and many areas in Chemistry. With regard to understanding the thermodynamics of PCMs, future R&D should extend the basis of information. Specifically, investigations should cover more homologous series, as well as similar groups of materials (e.g., the salts LiF, LiCl, LiBr, etc.) that promise to reveal further trends and correlations. This also includes a deeper study of the metals from main groups and those not from main groups. Complementary MDS studies of these materials can lead to a deeper and more detailed understanding of the origins of the correlations.

### 5.4. Non-Pure Substances, Meaning Mixtures

Last, but not least, a main reason to limit this review to pure substances was that little R&D has been published on the use of entropy for the understanding of the thermodynamics of non-pure substances, meaning mixtures, as PCMs. Starting points could be salt hydrates, clathrate hydrates, or other material classes that promise some systematic correlation of properties among members of the class.

## Data Availability

Data sharing is not applicable.

## Abbreviations

The following abbreviations are used in this manuscript:

TES	Thermal Energy Storage
PCM	Phase Change Material
IUPAC	International Union of Pure and Applied Chemistry
AN	Atomic Number
PSE	Periodic System of Elements
MDS	Molecular Dynamic Simulation
PE	Polyethylene
